# Supplementary material for: siVAE: interpretable deep generative models for single-cell transcriptomes
Source: Genome Biol. 2023 Feb 20;24:29. doi: 10.1186/s13059-023-02850-y (PMC9940350; doi:10.1186/s13059-023-02850-y)
Supplement: Supplementary file 1 — Additional file 1: Figures S1-S25, Tables S1-S3, and Supplementary Notes 1-2. [file 13059_2023_2850_MOESM1_ESM.pdf]

Supplementary materials for

siVAE: interpretable deep generative models for single cell transcriptomes

Yongin Choi[1,2], Ruoxin Li[2,3], Gerald Quon[1,2,4,*]

[1]Biomedical Engineering Graduate Group, [2]Genome Center, [3]Graduate Group in Biostatistics, [4]Department of Molecular and Cellular Biology, University of California, Davis, Davis, CA

*To whom correspondence should be addressed: gquon@ucdavis.edu

Table of Contents

Fig. S1. Non-linear dimensionality reduction methods generate cell embedding spaces in which cells of the same cell type cluster more tightly 3

Fig. S2. Clustering performance of cell embeddings are consistent across clustering metrics 4

Fig. S3. Clustering accuracy of cell embeddings are consistent across the different cell types ... 5

Fig. S4. Negative log likelihoods achieved by different methods on the fetal liver atlas dataset 6

Fig. S5. Increasing the weight of the siVAE interpretability term leads to lower performance .. 7

Fig. S6. siVAE achieves classification accuracy comparable to VAE on imaging datasets 8

Fig. S7. Classification experiments on three imaging datasets 9

Fig. S8. Intuitive visualization of feature embeddings using MNIST digit images 10

Fig. S9. Visualization of feature attributions across methods 11

Fig. S10. Runtimes of siVAE and feature attributions are consistent across batches 12

Fig. S11. Co-expressed gene modules cluster in the feature embedding space 13

Fig. S12. Disconnected genes cluster towards the origin only when the interpretability term has non-zero weight 14

Fig. S13. Clustering of marker genes based on CellTypist markers requires higher hierarchy of cell types 16

Fig. S14. Marker genes in CellTypist for different cell types overlap strongly 15

Fig. S15. High correlation between ground truth degree centrality (DC) and siVAE-estimated reconstruction accuracy 17

Fig. S16. siVAE predicts genes with higher degree centrality compared to other methods 18

Fig. S17. Both dimensionality reduction based approaches and explicit GCN inference based approaches predict neighborhood genes that equally explain the expression of query genes 19

Fig. S18. Overlap between neighborhood genes identified by different methods 20

Fig. S19. Cell lines separate according to differential efficiency in the cell line embedding space....................21

Fig. S20. Correlation between mitochondrial genes' degree centrality and neuronal differential efficiency is not driven by change in average expression levels....................22

Fig. S21. Number of edges between mitochondrial genes is significantly smaller for cell lines with higher efficiency....................23

Fig. S22. Single variant testing does not detect any associations between mitochondrial variants and differentiation efficiency....................24

Fig. S23. Schematic of the siVAE neural network....................25

Fig. S24. Choice of method for reducing the number of inputs to the siVAE feature-wise encoder-decoder network is robust to reduction method and number of reduced dimensions26

Fig. S25. Train/test losses for LDVAE and scVI....................27

Table S1: Architectures used for training on different datasets....................28

Table S2: Metadata on the datasets used in our study....................29

Table S3. Mapping of marker gene sets from MSigDB that were matched to the cell type labels in the fetal liver dataset.....................30

Supplementary Note 1. Visual validation of feature attribution from the imaging dataset....31

Supplementary Note 2. Mitochondrial variants for iPSC cell lines....................32

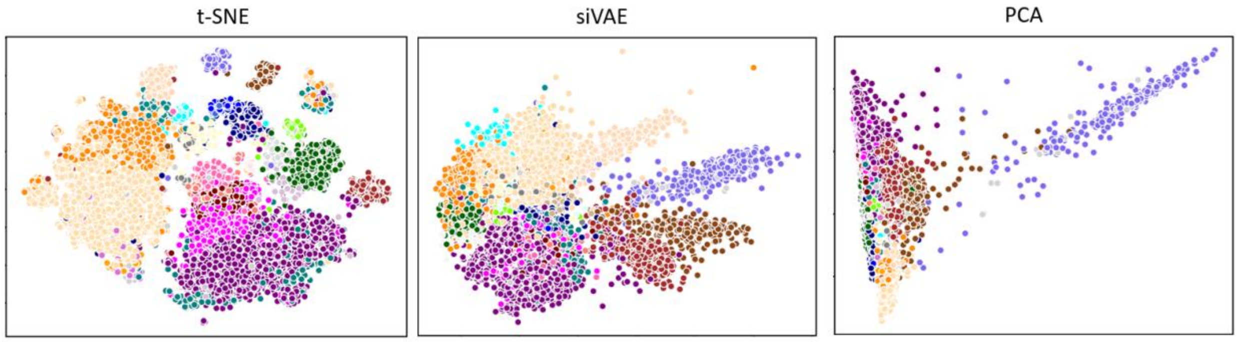

Fig. S1. Non-linear dimensionality reduction methods generate cell embedding spaces in which cells of the same cell type cluster more tightly. Scatterplots show embedding spaces generated using t-SNE, siVAE, and PCA trained on the fetal liver atlas dataset. Cells are colored based on cell type.

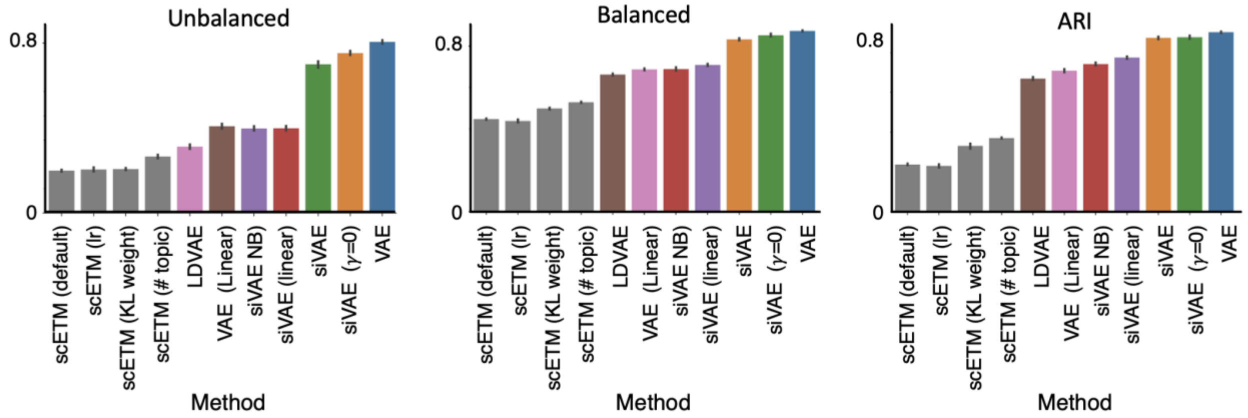

Fig. S2. Clustering performance of cell embeddings are consistent across clustering metrics. Bar plots indicate clustering performance based on either nearest neighborhood classification or ARI. For “All”, all cell types were used without considering differences in the number of cell types (unbalanced accuracy), whereas "Balanced” measures accuracy normalized by size for each cell type.

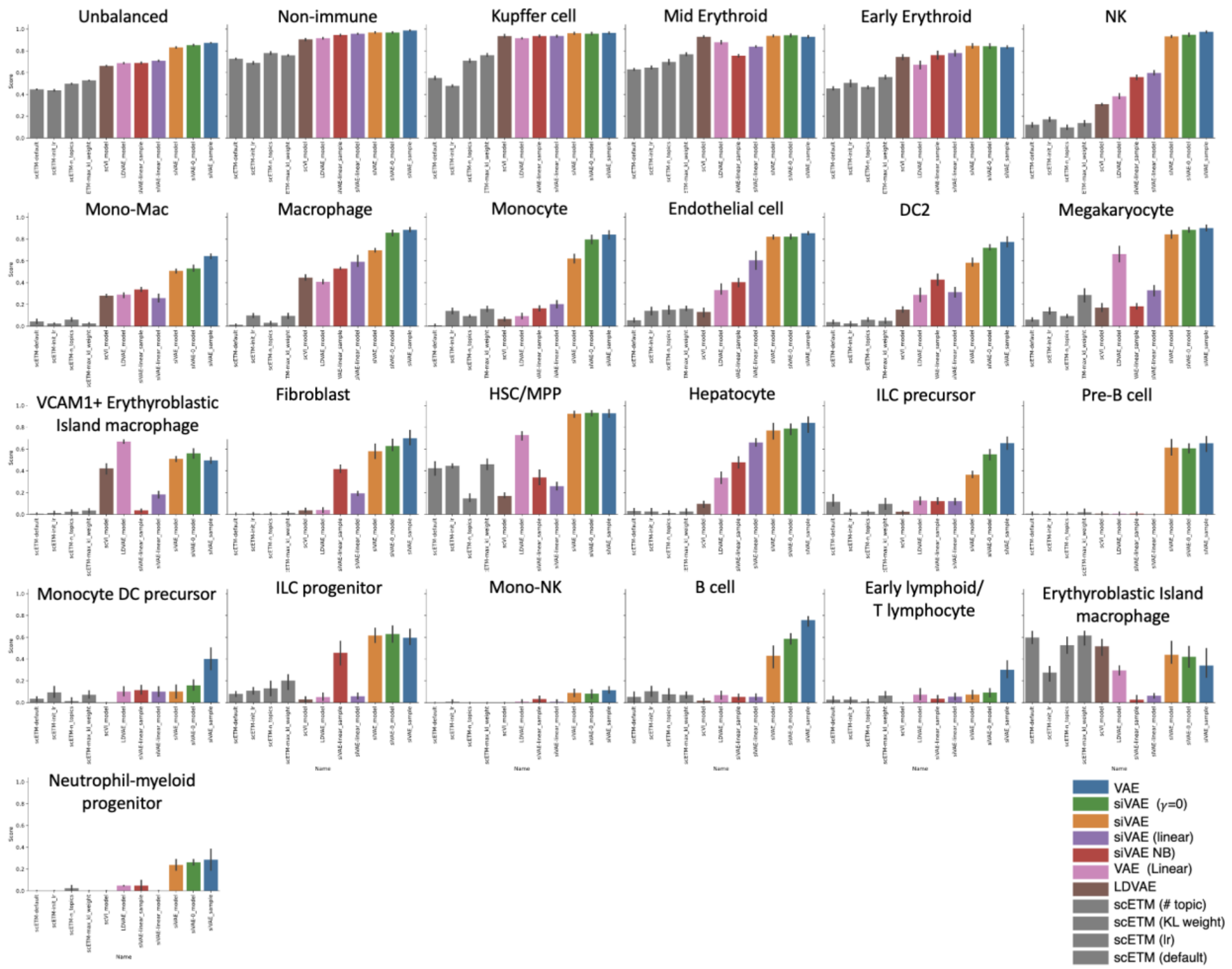

Fig. S3. Clustering accuracy of cell embeddings are consistent across the different cell types. Bar plots indicate clustering accuracy for each cell type in the FetalLiverAtlas dataset, ordered by decreasing number of cells.

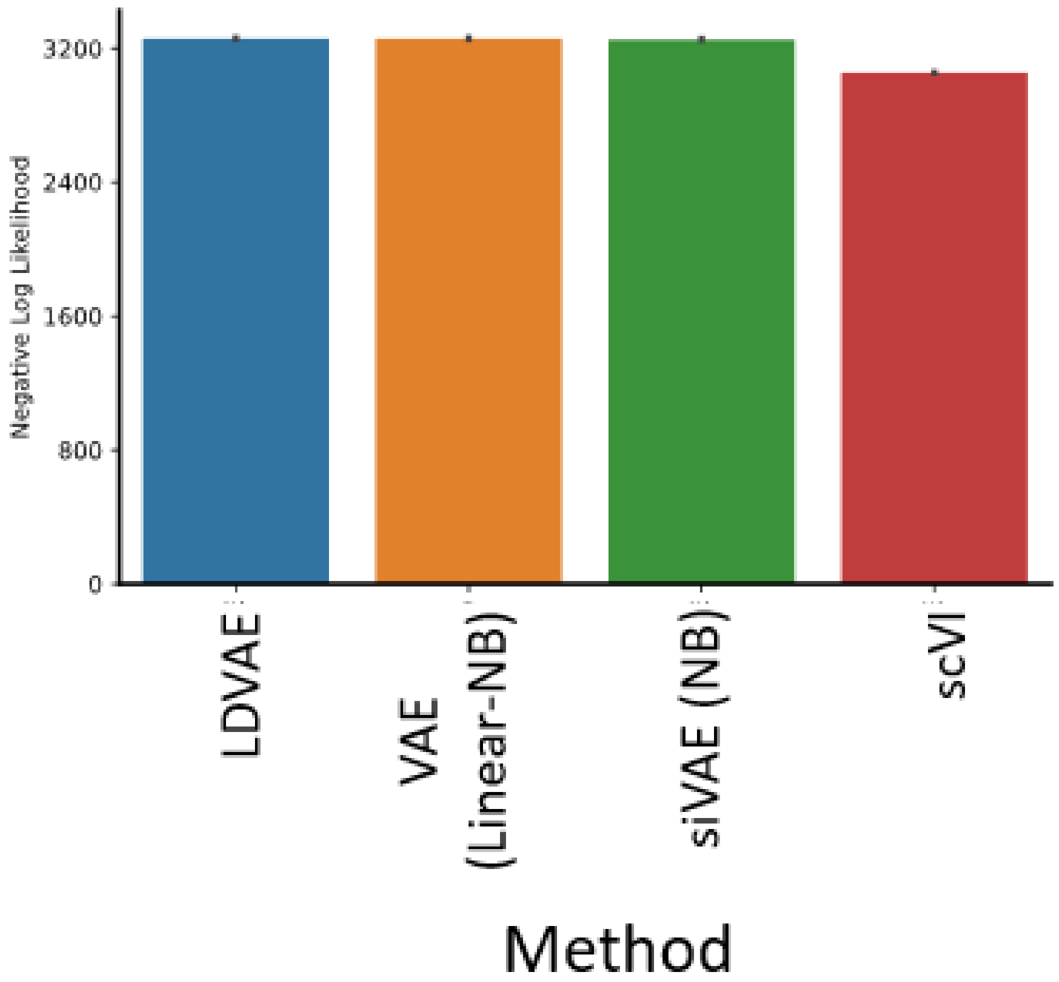

Fig. S4. Negative log likelihoods achieved by different methods on the fetal liver atlas dataset. Bar plot indicates the negative log likelihood (nll) for different models that use a negative binomial distribution as the output layer of the neural network.

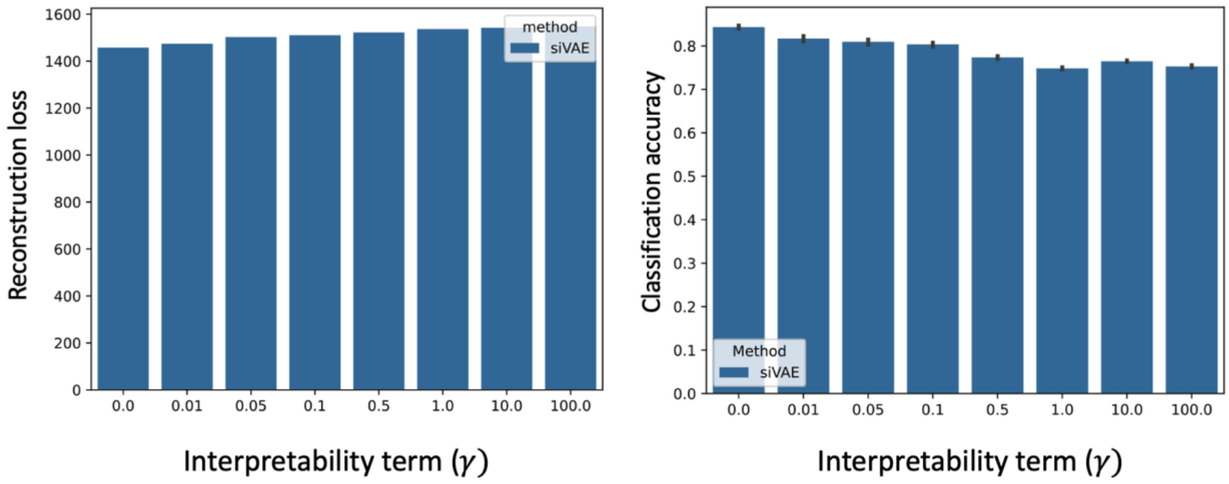

Fig. S5. Increasing the weight of the siVAE interpretability term leads to lower performance. We trained siVAE with varying weight on the interpretability term (γ) on the fetal liver atlas dataset. The bar plot shows (**left**) reconstruction loss and (**right**) clustering accuracy of the embedding space based on cell type labels, measured with a k-nearest neighbor classifier.

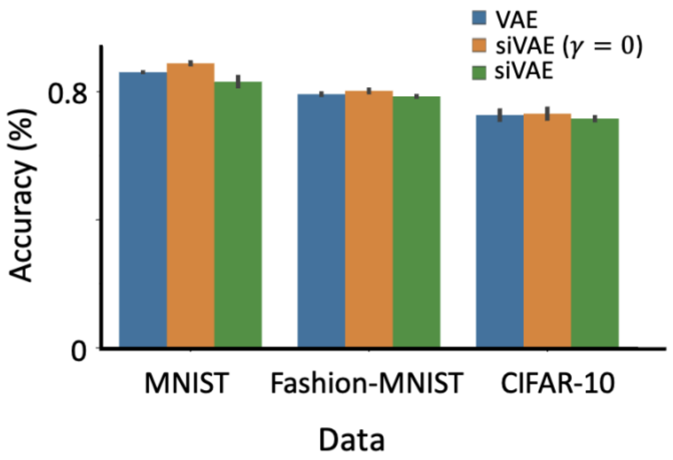

Fig. S6. siVAE achieves classification accuracy comparable to a canonical VAE on imaging datasets. Bar plot indicating classification accuracy on three imaging datasets. Each model was trained with an imaging dataset, and clustering accuracy of the embedding space based on image label was measured with k-nearest neighbors.

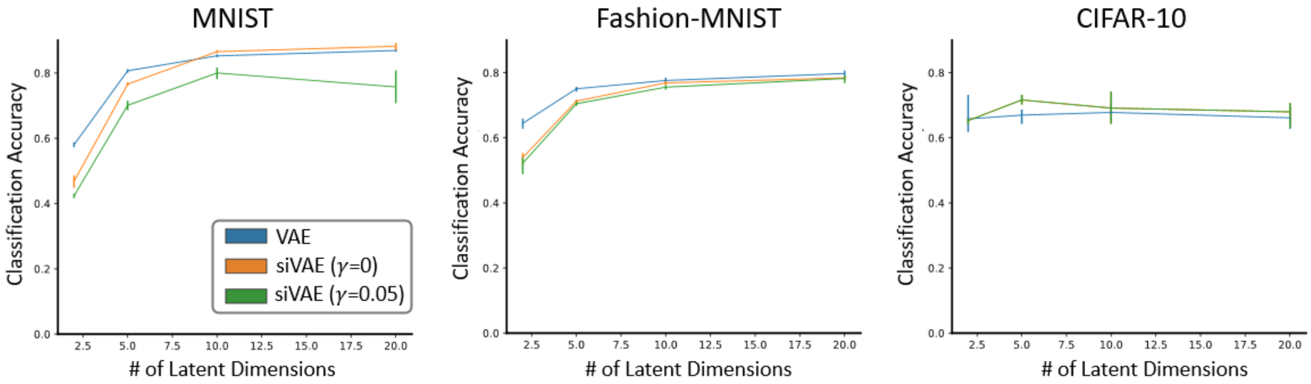

Fig. S 7. Classification experiments on three imaging datasets. Line plots indicate classification accuracy of methods trained on three imaging datasets, while varying the number of embedding dimensions. Classification was performed on the embeddings of each model using k-nearest neighbors in a 5-fold cross validation framework.

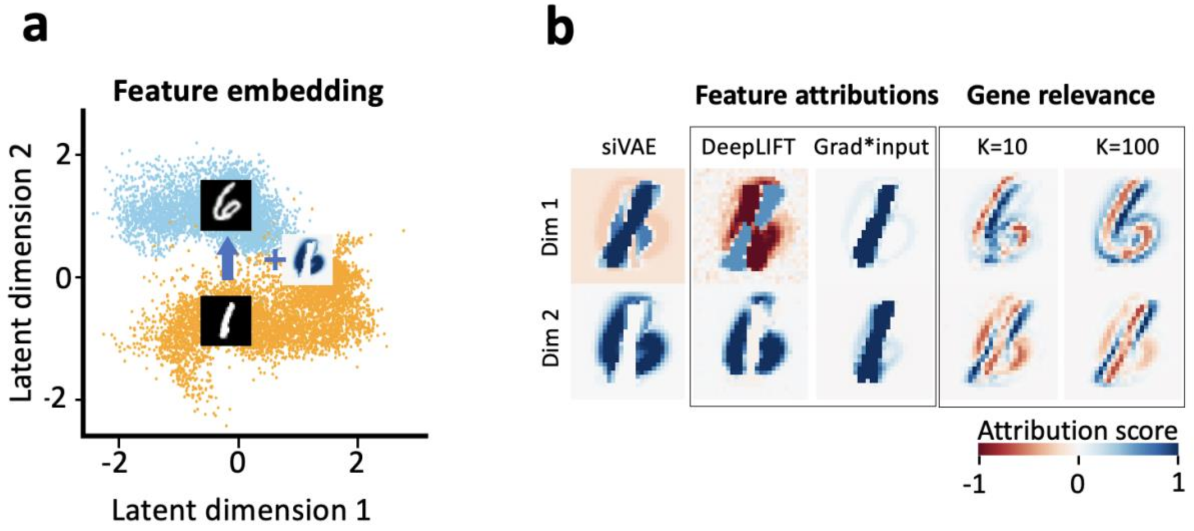

Fig. S8. Intuitive visualization of feature embeddings using MNIST digit images. To provide a visual comparison of feature attributions, we trained siVAE to perform dimensionality reduction on a subset of the MNIST dataset consisting of black and white digits. We focused on the digits 1 and 6 to ensure a human-visible separation of digits along individual embedding dimensions. (**a**) Scatterplot of feature embeddings inferred by siVAE trained on the one and six digits from the MNIST dataset. (**b**) Visualization of interpretations learned for each of the two dimensions (axes) from (a), for siVAE, Gene Relevance, DeepLIFT and grad*input.

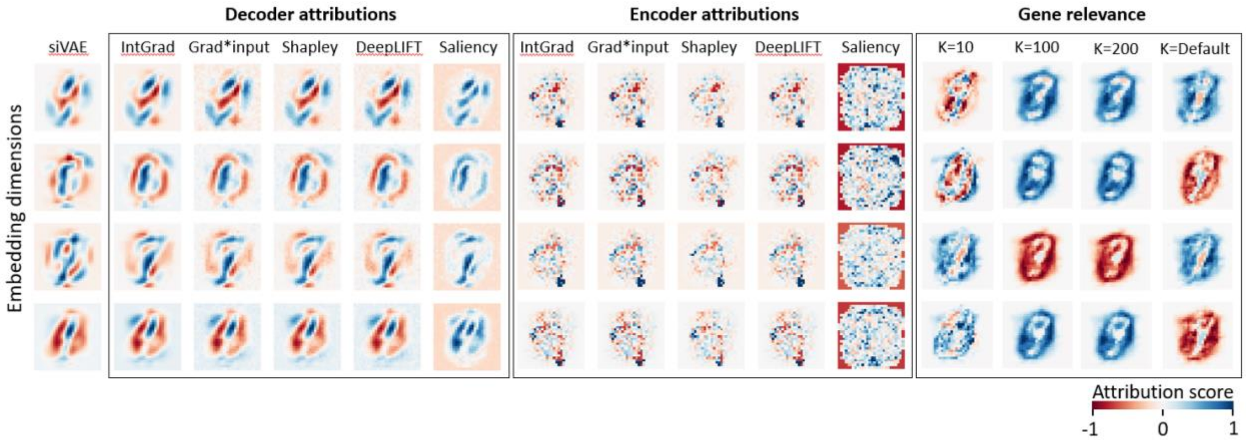

Fig. S 9. Visualization of feature attributions across methods. Visualization of feature attributions (or embeddings in the case of siVAE) for different methods when trained on the MNIST dataset with all digits. Individual images represent the feature attributions (or embeddings for siVAE) for one embedding dimension. Attribution score represents the contribution of individual features (pixel) to each embedding dimension. Feature attribution methods and gene relevance scores were computed on the trained siVAE model to make them comparable. siVAE interpretations are in better agreement with feature attribution methods (median Spearman $\rho = 0.89$, p=1.07e-11) compared to Gene Relevance (median Spearman $\rho = 0.12$, p=0.14).

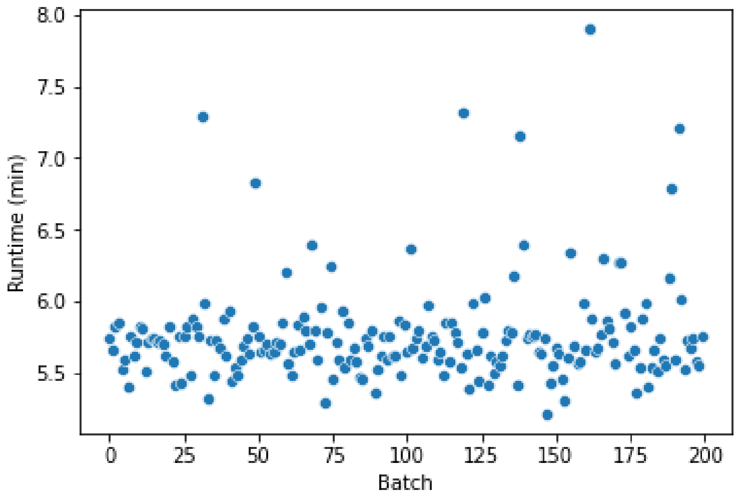

Fig. S10. Runtimes of siVAE and feature attributions are consistent across batches. Scatterplot where each point is a batch forward pass through the siVAE model, followed by the feature attribution operation. The y-axis is the execution time (mean=5.76 mins, std dev=0.35 mins), and the x-axis is the batch number.

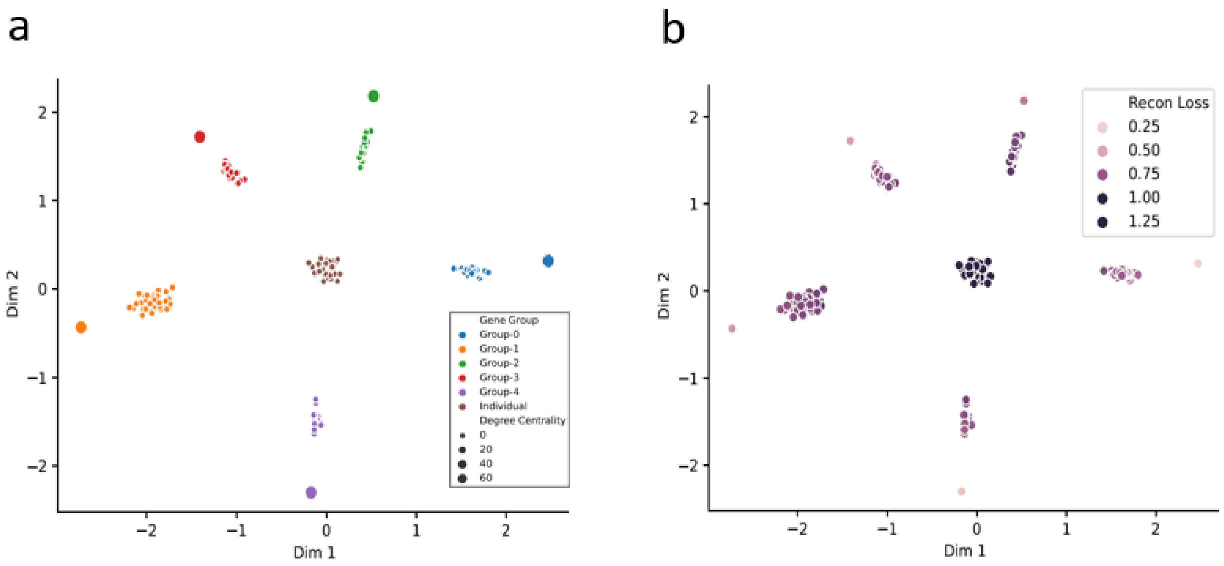

Fig. S11. Co-expressed gene modules cluster in the feature embedding space. Scatter plots show the feature embeddings of siVAE when trained on a dataset simulated from a hypothetical genome containing 300 genes, and in which the underlying gene network consists of five communities of co-regulated genes, and one group of disconnected nodes. (**a**) Nodes are colored based on which community they originate from. (**b**) Nodes are colored based on their reconstruction loss averaged across cells after training.

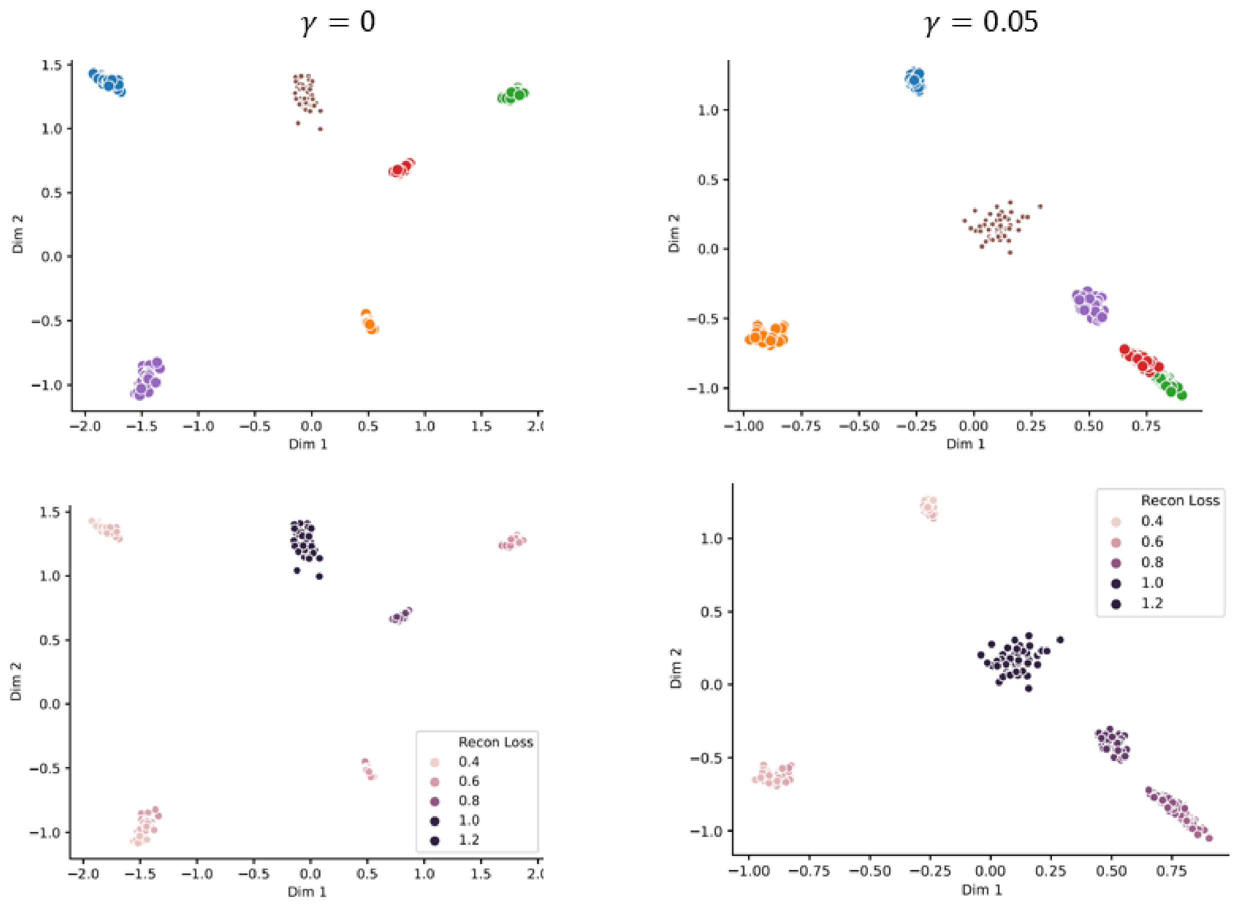

Fig. S12. Disconnected genes cluster towards the origin only when the interpretability term has non-zero weight. Scatter plots show the feature embeddings of siVAE when trained on a dataset simulated from a hypothetical genome containing 300 genes, and in which the underlying gene network consists of five communities of co-regulated genes, and one group of disconnected nodes. Scatterplots show the feature embeddings of siVAE with ($\gamma = 0.05$) and without ($\gamma = 0$) the interpretability term. Top row: nodes are colored based on which community they belong to. Bottom: nodes are colored by reconstruction error after training.

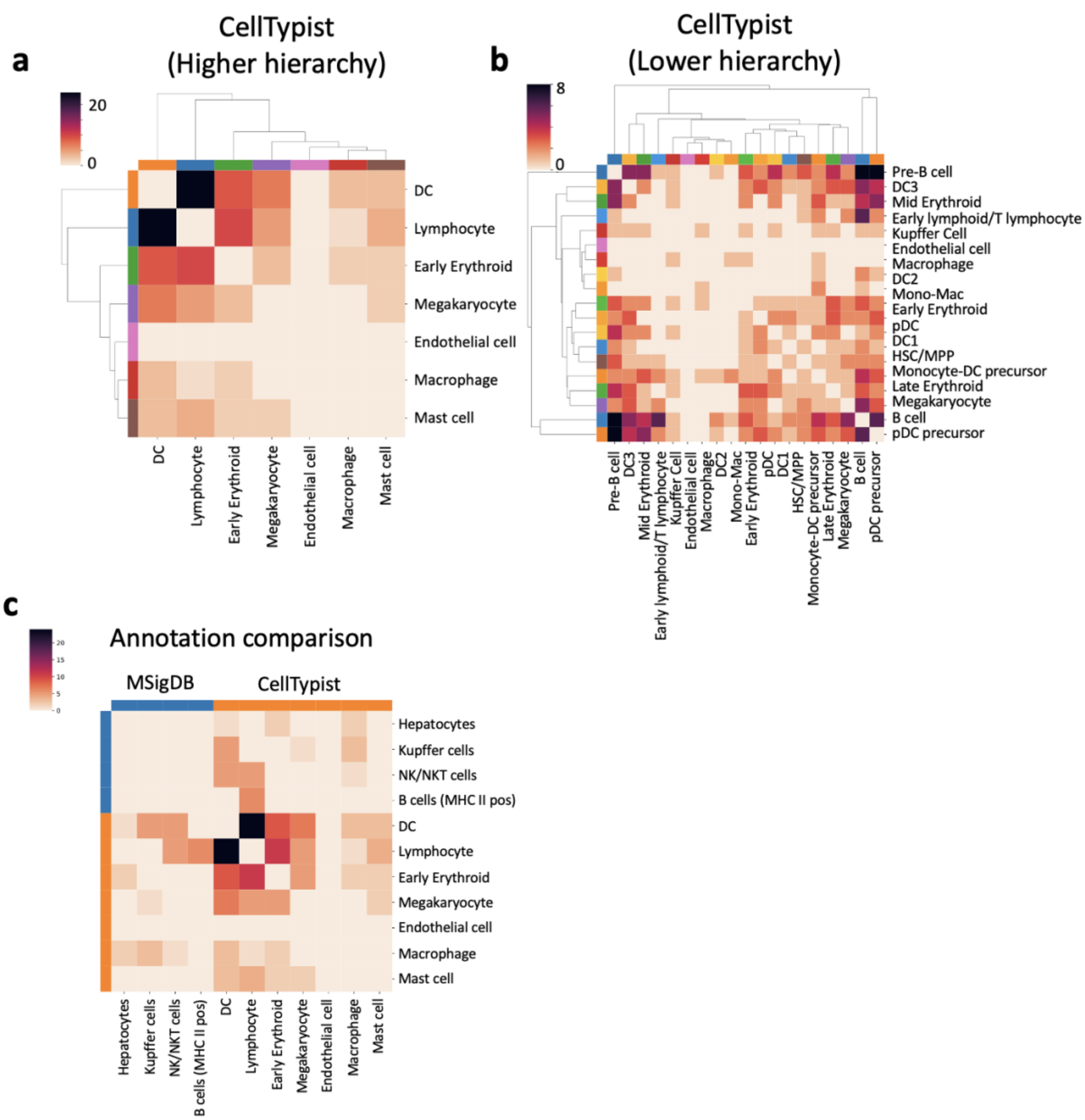

Fig. S13. Marker gene sets for different cell types defined in CellTypist overlap strongly. Heatmap indicates the overlap in marker gene sets between pairs of cell types, when using CellTypist annotations at the **(a)** higher hierarchy or **(b)** lower hierarchy. Additionally, overlap between MSigDB marker gene sets and CellTypist (higher hierarchy) gene sets are shown in **(c); Table S3** indicates how we combined MSigDB sets to produce marker gene sets.

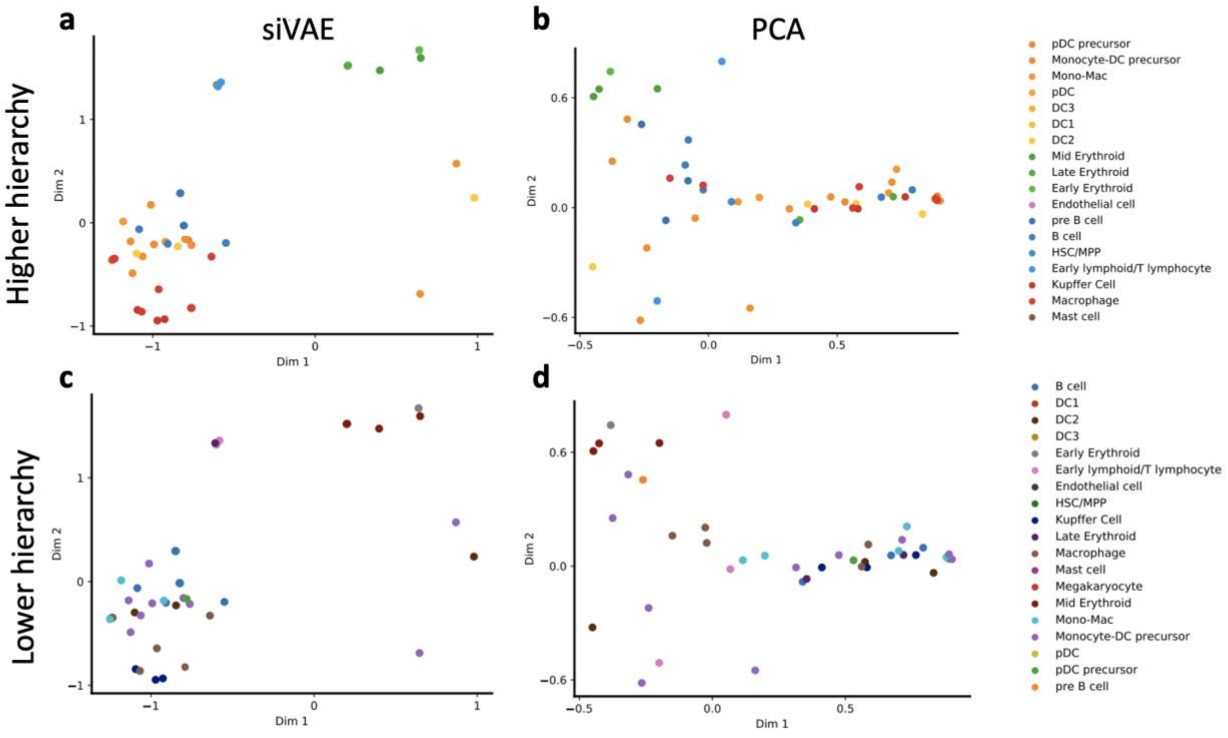

Fig. S14. Clustering of marker genes based on CellTypist markers is more pronounced using the higher hierarchy of cell types compared to the lower hierarchy of cell types. Scatter plot shows the feature embeddings of siVAE and PCA trained on the fetal liver dataset, where each point represents a marker gene. **(a)** Scatterplot of siVAE feature embeddings, where colors are based on the higher hierarchy cell types. **(b)** Scatterplot of PCA loadings of genes, where colors are based on the higher hierarchy cell types. **(c)** Scatterplot of siVAE feature embeddings, where colors are based on the lower hierarchy cell types. **(d)** Scatterplot of PCA loadings of genes, where colors are based on the lower hierarchy cell types.

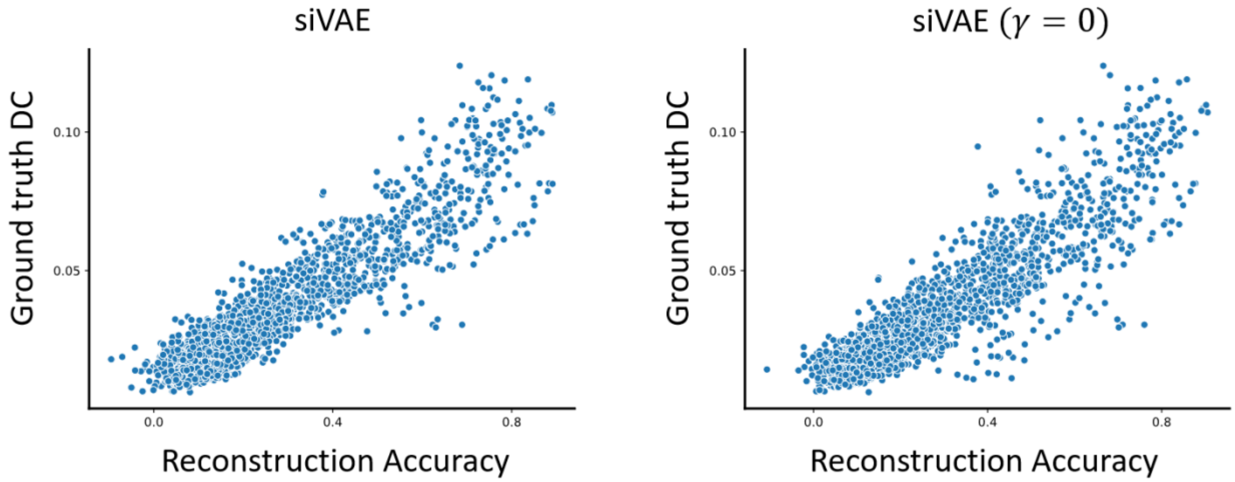

Fig. S15. High correlation between ground truth degree centrality (DC) and siVAE-estimated reconstruction accuracy. Scatter plot shows the correlation between predicted degree centrality (measured as reconstruction accuracy) and ground truth degree centrality for siVAE with and without ($\gamma = 0$) the interpretability term.

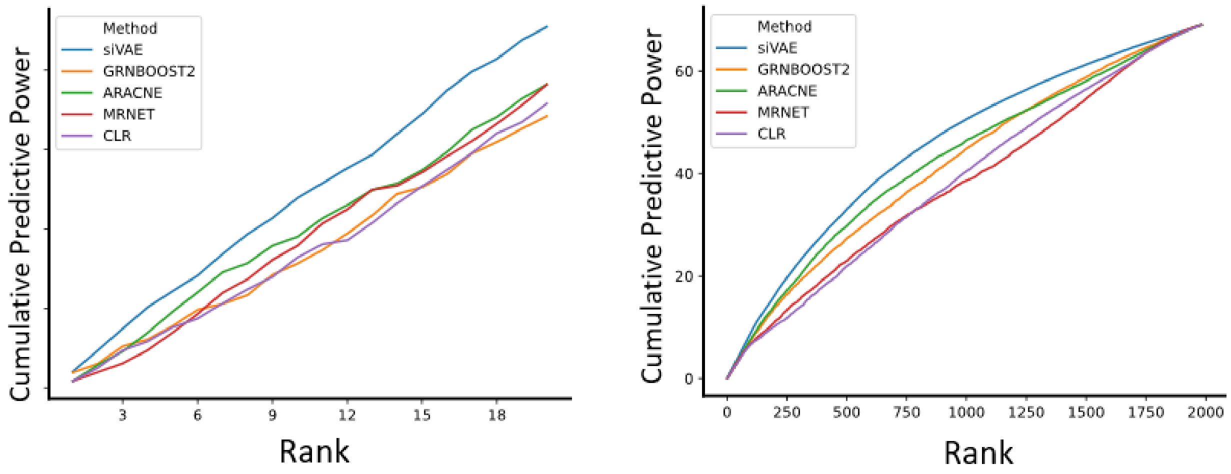

Fig. S16. siVAE predicts genes with higher ground truth degree centrality compared to other methods. Line plot indicates the cumulative ground truth degree centrality of the (**a**) top 20 and (**b**) top 2000 genes ranked in decreasing order of largest predicted degree centrality by each method.

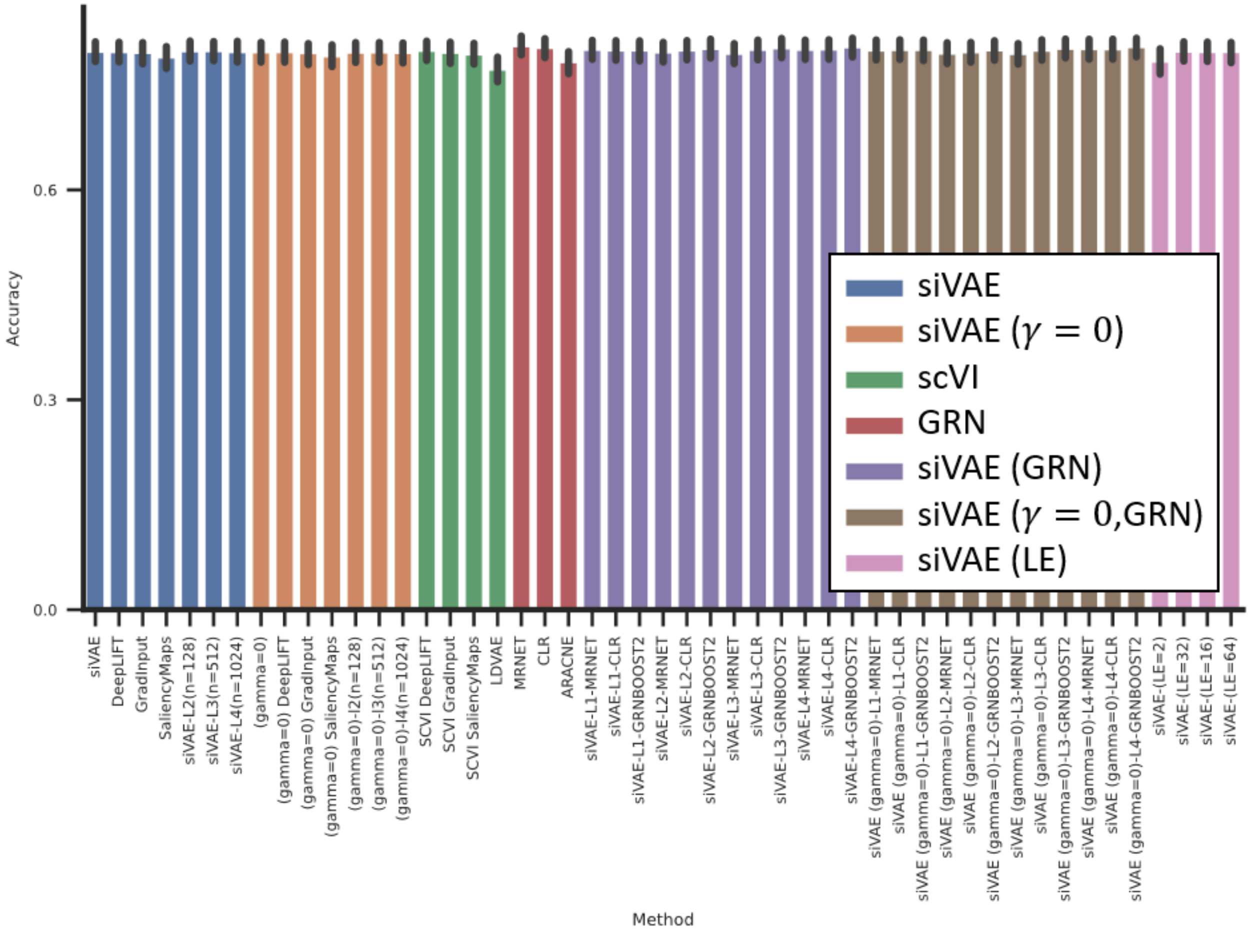

Fig. S17. Both dimensionality reduction based approaches and explicit GCN inference based approaches predict neighborhood genes that equally explain the variance in expression of query genes. Bar plot indicates the prediction accuracy (% of variance explained) of the neighborhood gene sets when predicting each query gene, averaged over the 152 query genes with highest predicted degree centrality across tested methods. Blue bars denote methods based on siVAE with the interpretability term, and orange bars denote methods based on siVAE without interpretability term. Green bars denote methods based on applying feature attribution to scVI. Red bars indicate GCN inference based methods. Purple and brown bars denote approaches where GRN inference methods were applied to data sampled from a siVAE model trained on the original dataset, with and without the interpretability term respectively. Finally, pink bars denote methods based on siVAE with varying numbers of embedding dimensions.

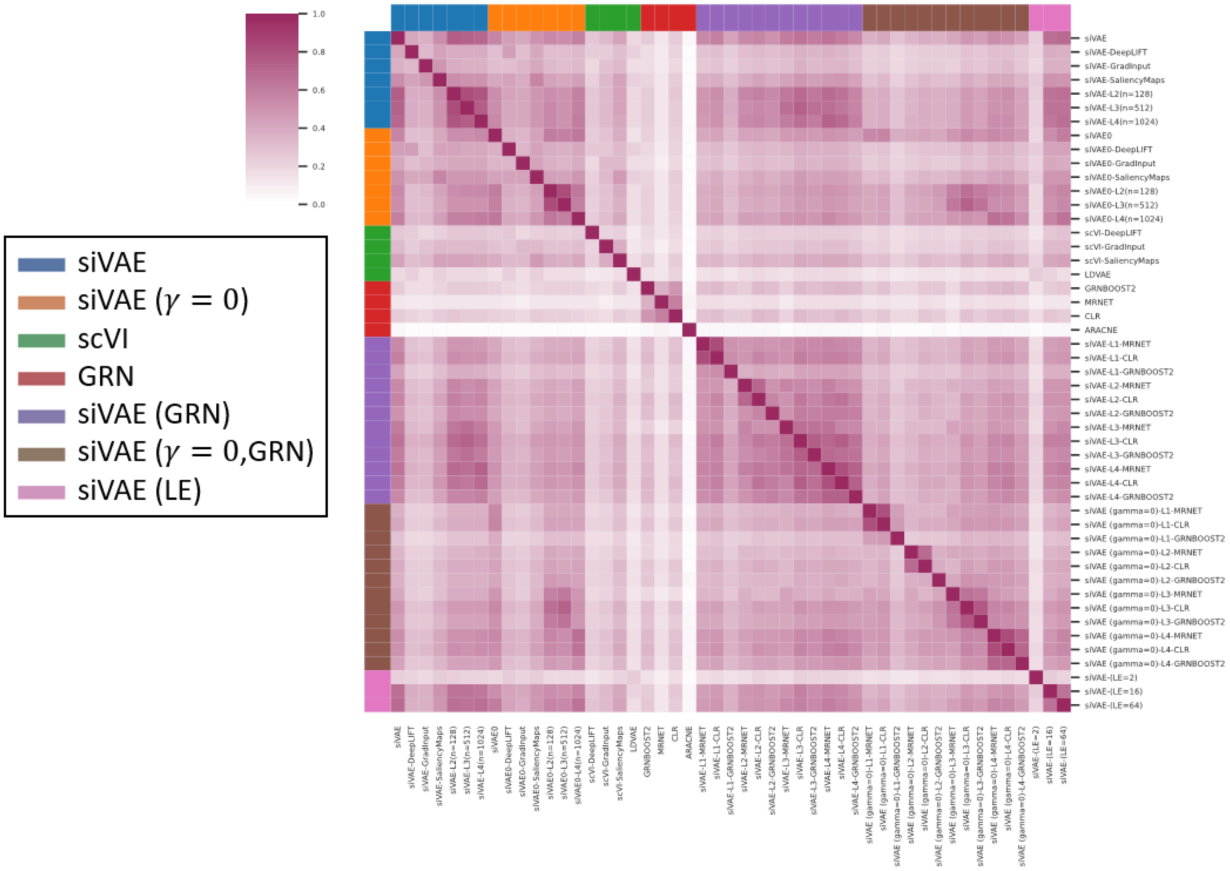

Fig. S18. Overlap between neighborhood genes identified by different methods. Heatmap indicates the Jaccard index quantifying the overlap between the neighborhood genes detected by each method.

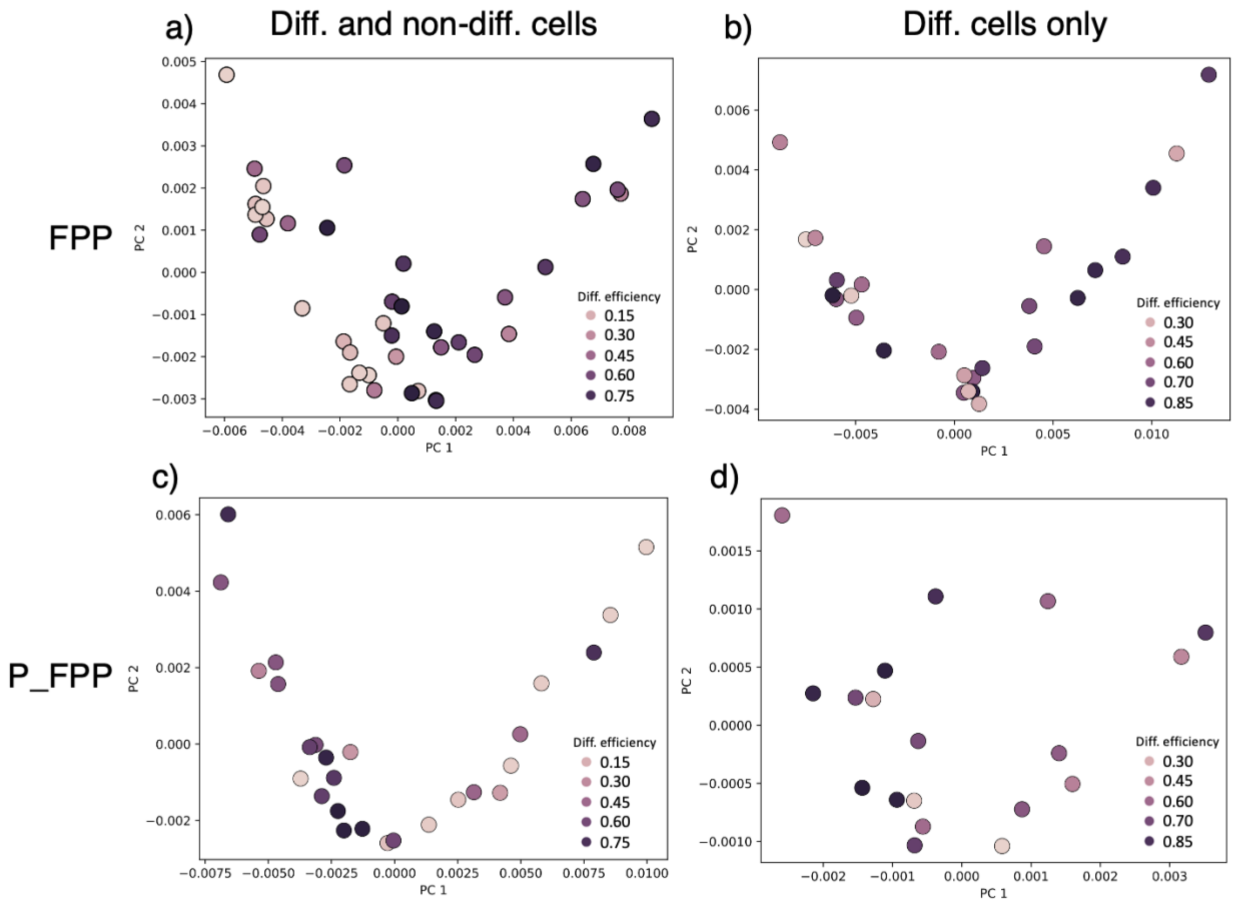

Fig. S19. Cell lines separate by differential efficiency in the cell line embedding space. Scatter plots of embeddings of cell line-specific gene co-expression networks implicitly learned by siVAE, and colored based on neuronal differentiation efficiency. (**a**) siVAE was trained on the FPP cell type from all donor lines. PC-1 is strongly correlated with efficiency (Spearman $\rho = 0.62$,P=3.0x10^{-5}). (**b**) siVAE was trained on the FPP cell type from only those donor lines that were successfully differentiated. PC-1 is still strongly correlated with efficiency (Spearman $\rho = 0.59$,P=4.8x10^{-4}). (**c**) siVAE was trained on the P_FPP cell type from all donor lines. PC-1 is strongly correlated with efficiency (Spearman $\rho = 0.55$,P=4.2x10^{-3}). (**d**) siVAE was trained on the P_FPP cell type from only those donor lines that were successfully differentiated. PC-1 is still strongly correlated with efficiency (Spearman $\rho = 0.53$, P=0.019).

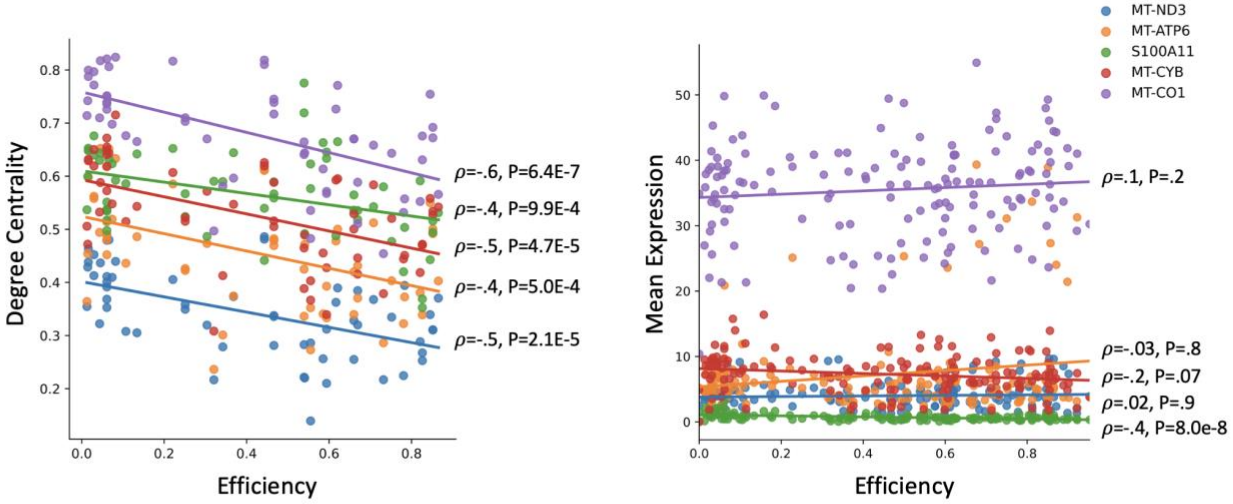

Fig. S20. Correlation between mitochondrial genes' degree centrality and neuronal differential efficiency is not driven by change in average expression levels. (**left**) Scatter plot showing correlation between each MT gene's degree centrality (y-axis) versus efficiency (x-axis). Individual points represent an MT gene for a specific cell line; points are colored based on the mitochondrial gene identity. (**right**) Same as left, but the y-axis represents mean expression of a MT gene in a cell line.

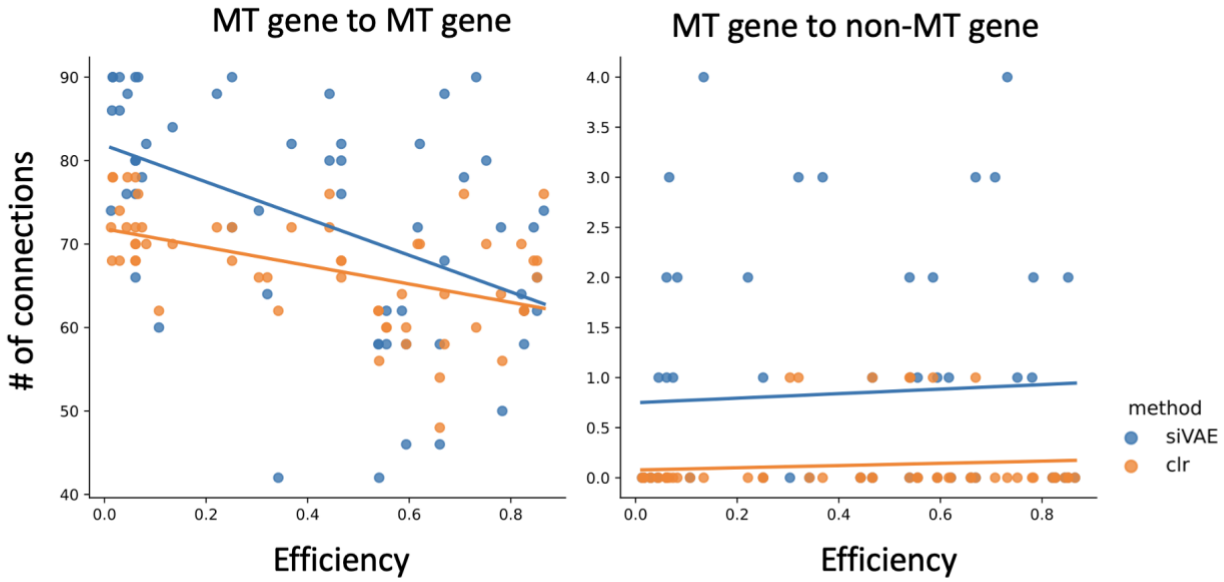

Fig. S21. Number of edges between mitochondrial genes is significantly smaller for cell lines with higher efficiency. (**left**) Scatter plot showing negative correlation between the number of edges between mitochondrial genes (y-axis) versus differentiation efficiency (x-axis). Individual points represent a gene for a specific cell line. The number of edges per cell line was calculated from the gene network inferred from either siVAE or CLR. (**right**) Same as left, but the y-axis represents the number of edges between one mitochondrial gene and one non-mitochondrial gene.

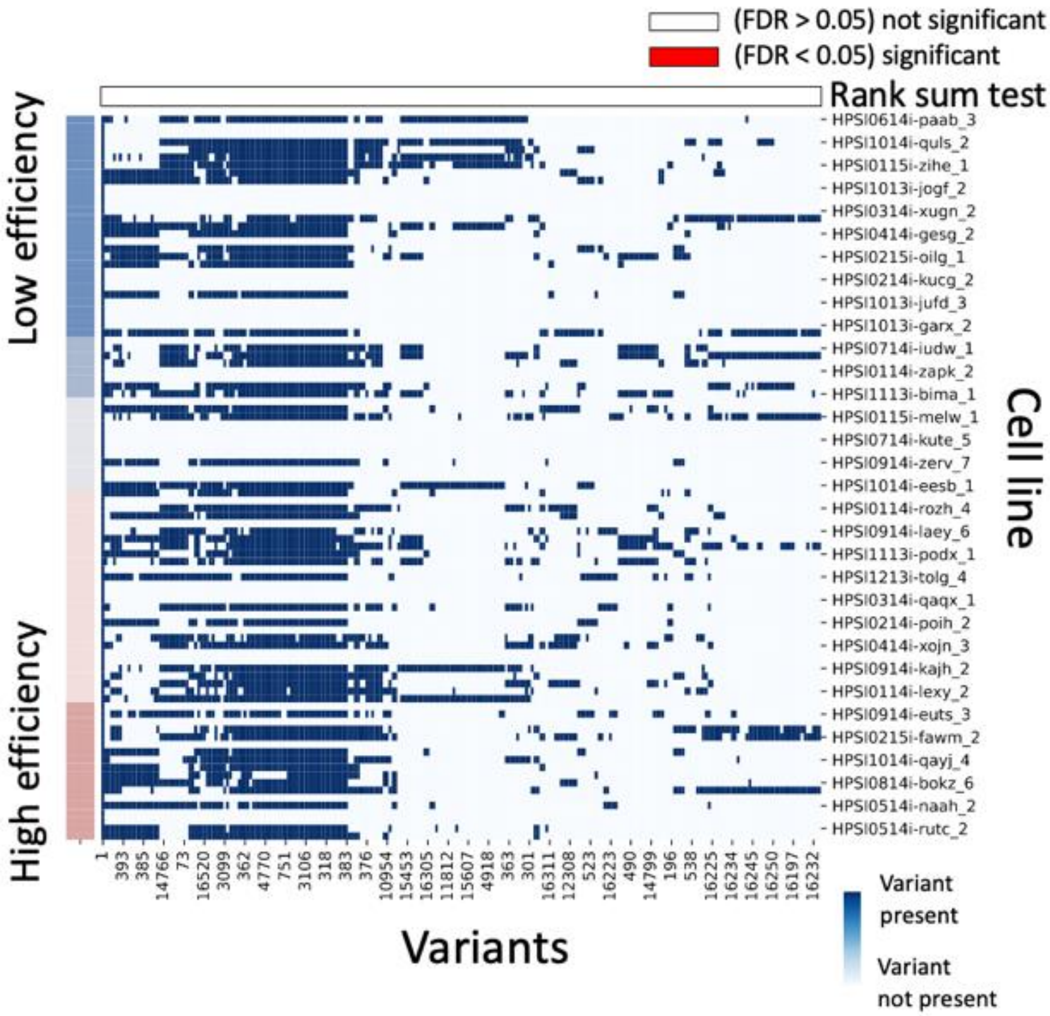

Fig. S22. Single variant testing does not detect any associations between mitochondrial variants and differentiation efficiency. Heatmap indicates the presence of a variant in a cell line. Cell lines are sorted according to their neuronal differential efficiency. Hierarchical clustering is performed on the columns for variants. Column colorbar shows the result of Wilcox rank sum tests.

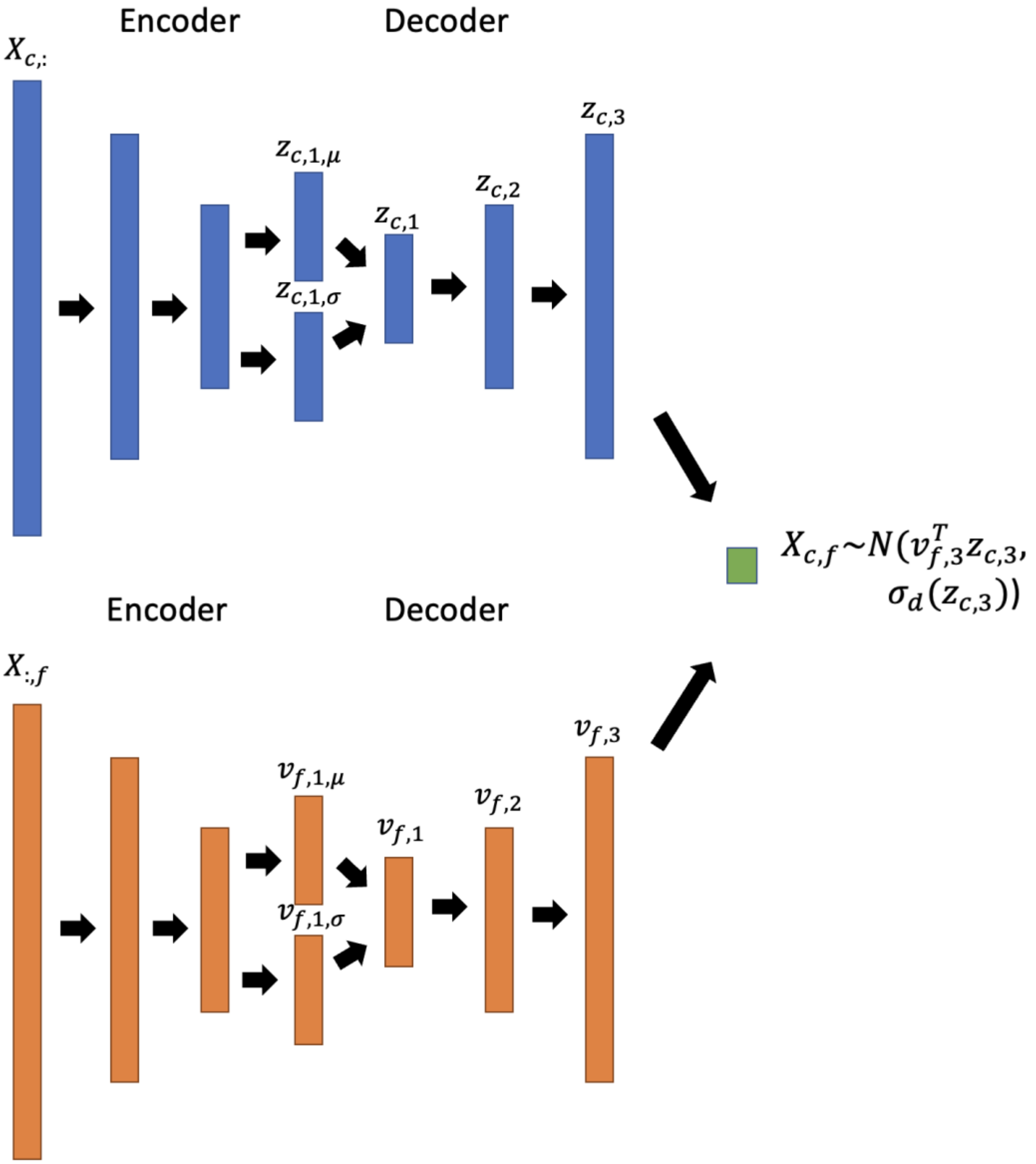

Fig. S23. Schematic of the siVAE neural network. Neural network setup and operations for siVAE including the cell-wise encoder-decoder on top in blue, and the feature-wise encoder-decoder on bottom in orange. Layers are labeled with variables consistent with the Methods section of the main text.

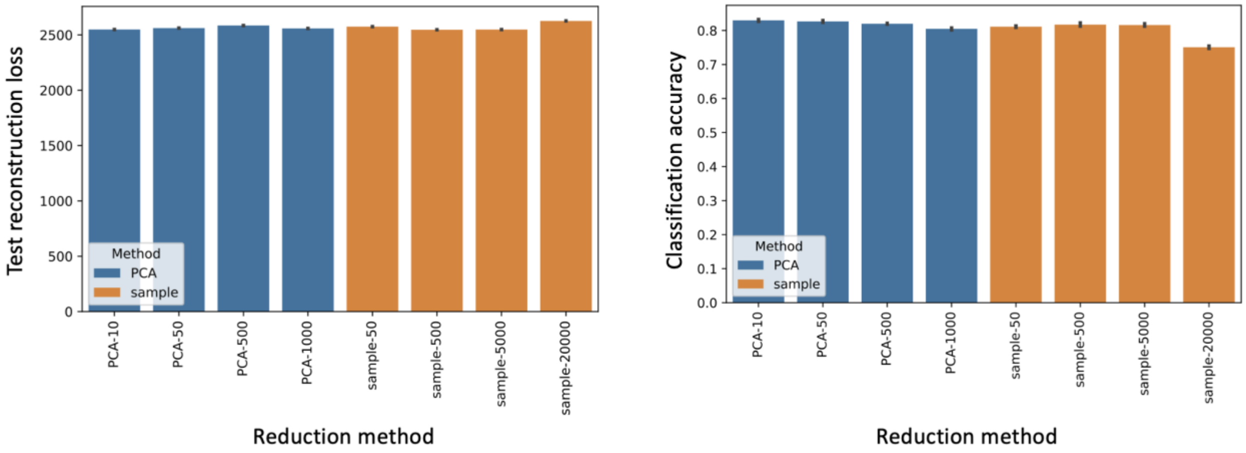

Fig. S24. Choice of method for reducing the number of inputs to the siVAE feature-wise encoder-decoder network is robust to reduction method and number of reduced dimensions. Two reduction methods, PCA and downsampling, were used to reduce the input dataset size. We varied the number of PCs for PCA, and the number of retained features for the downsampling approach; the numbers chosen are indicated in the method name. (**top**) Bar plot showing test reconstruction loss of siVAE. (**bottom**) Bar plot showing classification accuracy of siVAE.

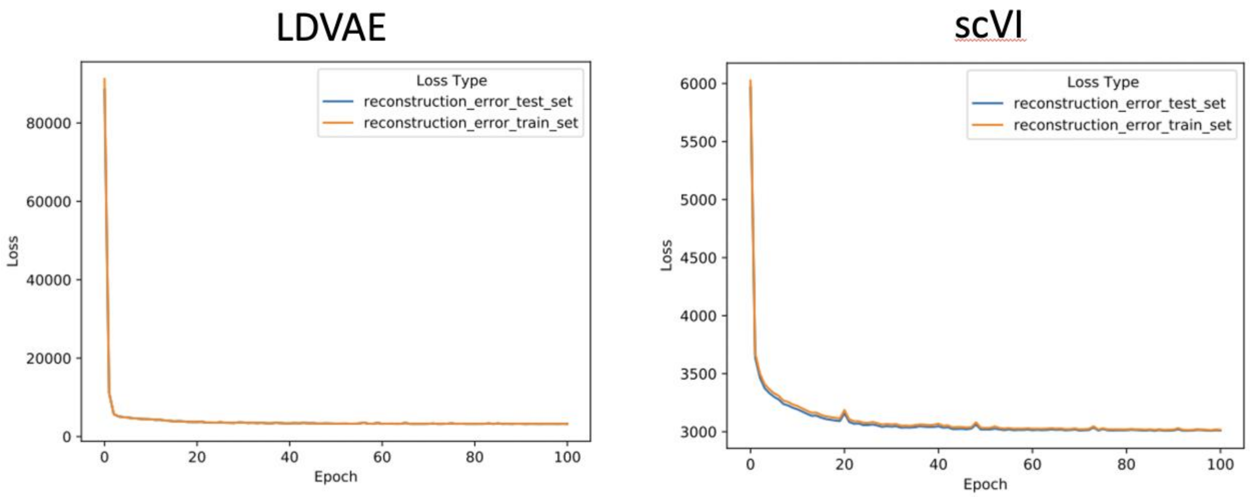

Fig. S25. Train/test losses for LDVAE and scVI. Line plot of train and test losses for LDVAE and scVI as a function of epoch, confirming convergence of the models.

Dataset name	Encoder architecture	# of latent dimension	Decoder architecture
MNIST	512-128	{2,5,10,20}	128-512
Fashion-MNIST	512-128	{2,5,10,20}	128-512
CIFAR-10	1024-512-128	{2,5,10,20}	128-512-1024
Fetal liver atlas	1024-512-128	{2,64}	128-512-1024
1.3 Million Brain Cells	2048-1024-512	{20,128,512}	512-1024-2048
scATAC-Seq	1024-512-128	{20}	128-512-1024
NeurDiff	1024-512-128	{32}	128-512-1024

Table S1: Architectures used for training on different datasets. For the encoder and decoder architectures, each number separated by a dash indicates the number of nodes for a single layer. # of latent dimensions indicates the set of all numbers of latent dimensions that were tested throughout the experiments.

Dataset name	# Class	# Sample	# Feature
MNIST	10	60,000	784
Fashion-MNIST	10	60,000	784
CIFAR-10	2	10,000	3,072
Fetal liver atlas	40	100,000	2,000
1.3 Million Brain Cells	NA	1,308,421	27,998
scATAC-Seq	NA	8,000	244,544
NeurDiff (FPP)	41	109,483	3,362
NeurDiff (P_FPP)	27	85961	3,308

Table S2: Metadata on the datasets used in our study.

MSigDB meta-marker set	FetalLiverAtlas cell type	MSigDB gene sets
Hepatocytes	Hepatocytes	Aizarani_Liver_C11_Hepatocytes_1<br>Aizarani_Liver_C30_Hepatocytes_4<br>Aizarani_Liver_C17_Hepatocytes_3<br>Aizarani_Liver_C14_Hepatocytes_2
Kupffer cells	Kupffer cells	Aizarani_Liver_C6_Kupffer_Cells_2<br>Aizarani_Liver_C2_Kupffer_Cells_1<br>Aizarani_Liver_C31_Kupffer_Cells_5<br>Aizarani_Liver_C25_Kupffer_Cells_4<br>Aizarani_Liver_C23_Kupffer_Cells_3
B cells (MHC II Positive)	Pro B cell<br>Pre B cell<br>Pre pro B cell	Aizarani_Liver_C34_MHC_II_pos_B_cells<br>Aizarani_Liver_C38_Resident_B_cells_3<br>Aizarani_Liver_C8_Resident_B_cells_1<br>Aizarani_Liver_C22_Resident_B_cells_2
NK/NKT cells	NK<br>Mono-NK<br>Mac NK	Aizarani_Liver_C28_NK_NKT_cells_6<br>Aizarani_Liver_C1_NK_NKT_cells_1<br>Aizarani_Liver_C12_NK_NKT_cells_4<br>Aizarani_Liver_C5_NK_NKT_cells_3<br>Aizarani_Liver_C3_NK_NKT_cells_2<br>Aizarani_Liver_C18_NK_NKT_cells_5

Table S3. Mapping of marker gene sets from MSigDB that were matched to the cell type labels in the fetal liver dataset. Cell type category represents our higher-level grouping of the Aizarani cell types into larger categories for visualization.

Supplementary Note 1. Visual validation of feature attribution from the imaging dataset. We performed the same comparison on the MNIST imaging dataset as we did on Fetal Liver Atlas dataset by calculating feature attributions. With the imaging dataset, we were able to visualize the interpretation as an image, where contribution of individual features (pixel) is represented on a color scale (**Fig. S8**). We found that again siVAE interpretations agreed more strongly with the neural net attribution methods (median Spearman correlation of 0.48, P=2.4e-27) compared to Gene Relevance (median Spearman correlation of 0.35, P=0.10).

Supplementary Note 2. Mitochondrial variant association testing for iPSC cell lines. We tested the possibility that mitochondrial variants could be associated with differentiation efficiency. We obtained variant calls for the iPSC cell lines in NeurDiff dataset from the HipSci repository, then performed Wilcoxon rank sum test on single variants and gene-based burden testing on variants grouped by gene. However, there were neither single variants nor grouped variants that were significantly correlated with efficiency based on their adjusted P-value (**Supplementary Fig. S23**).
